# Nut consumption in chronic kidney disease: a systematic review

**DOI:** 10.3389/fnut.2025.1659516

**Published:** 2025-11-10

**Authors:** Taline Lazzarin, Raquel SimÃµes Ballarin, Paula Schmidt Azevedo, Barbara R. Cardoso, Marcos Ferreira Minicucci

**Affiliations:** 1 Internal Medicine Department, Medical School, São Paulo State University (Unesp), Botucatu, Brazil; 2 Department of Nutrition, Dietetics and Food, Monash University, Melbourne, VIC, Australia; 3 Victorian Heart Institute, Monash University, Clayton, VIC, Australia

**Keywords:** nuts, chronic kidney disease, nutrition, inflammation, oxidative stress, blood pressure, mortality

## Abstract

**Introduction:**

Nuts possess a beneficial nutritional profile with antioxidant and anti-inflammatory properties, potentially improving health outcomes in chronic kidney disease (CKD) patients. This systematic review evaluates the association between nut intake and lipid profile, inflammation, oxidative stress, blood pressure, and mortality in patients with CKD.

**Methods:**

A systematic search was performed in Medline, EMBASE, Scopus, CINAHL, and Cochrane Library databases on 27 May 2024 and repeated on 13 February 2025 without restrictions on date, language, or study type.

**Results:**

The search identified 2,521 records, with four studies (three trials, one cohort) involving 1,270 CKD patients meeting the inclusion criteria. The studies were heterogeneous in terms of intervention and biomarkers assessed, and all of them had moderate-to-high risk of bias. Overall the findings indicate positive effects of Brazil nuts and baru almond oil on oxidative stress and inflammation markers, while walnuts reduced LDL cholesterol and blood pressure while posing no risk regarding electrolytes balance. Further, the consumption of total nuts was associated with reduction in all-cause mortality.

**Conclusion:**

Our review indicates potential benefits for the inclusion of nuts in the diet of patients with CKD, but further studies are required to translate findings into practice.

**Systematic review registration:**

PROSPERO, Registration no CRD42024543880. https://www.crd.york.ac.uk/PROSPERO/view/CRD42024543880.

## 1 Introduction

Chronic kidney disease (CKD) is characterized by diminished kidney function, shown by a glomerular filtration rate of less than 60 mL/min per 1.73 m^2^, or markers of kidney damage, or both, present for a minimum of 3 months, causing negative health implications (1). Affecting approximately 850 million people worldwide, CKD is responsible for 41.5 million disability-adjusted life years and 1.43 million deaths annually (1, 2). Traditional risk factors for CKD development and progression include diabetes, hypertension, and obesity. However, non-traditional factors such as chronic inflammation, oxidative stress, endothelial dysfunction, uremic toxins, and gut dysbiosis also play a central role in its pathophysiology and prognosis. In particular, inflammation and oxidative stress contribute to vascular injury, endothelial dysfunction, and accelerated atherosclerosis, which markedly increase the risk of cardiovascular morbidity and mortality in this population (3).

Nutritional therapy is a key part of CKD management aiming at slowing disease progression, reducing albuminuria, and minimizing the harmful effects of uremic toxins (4). The National Kidney Foundation’s Kidney Disease Outcomes Quality Initiative (KDOQI) has provided evidence-based dietary guidelines for the management of kidney diseases since 1999, with great emphasis on an individualized approach to monitor protein intake, as well as control sodium, potassium and phosphorus circulating levels to prevent complications. Further, their most recent guidelines indicate that supplementing the diet of people with CKD with vitamins and trace elements does not improve outcomes and is not routinely recommended for these patients (4). Thus, more research focusing on nutritional interventions is needed to elucidate strategies with the potential to reduce inflammation and oxidative stress in CKD patients.

Nuts, described as dry fruits with an edible seed and a hard shell, are considered nutrient-dense foods due to their high concentration of vitamins, minerals, and bioactive compounds with redox action in addition to a high ratio of omega-3: omega-6 fatty acids (5–8). Peanuts, despite being botanically classified as legumes, have a similar nutritional profile as tree nuts and are thus commonly included in this group (9). The effects of nut intake have been associated with reduced risk of chronic diseases such as diabetes, cancer, cardiovascular diseases and cognitive impairment (10–14). Further, previous research has indicated the benefits of nuts on the oxidative stress and inflammation biomarkers in hemodialysis patients, indicating that this food group could serve as an affordable and manageable alternative for CKD management (15). Nonetheless, the effects of nut intake on CKD patients’ prognosis are still unclear. Further, some concerns have been raised regarding the high content of protein, phosphorus, and potassium present in nuts, since these nutrients are commonly restricted in the diet of CKD patients (4).

Therefore, this systematic review aims to synthesize current evidence on the association between nut consumption and health outcomes in patients with CKD, with a specific focus on mortality, lipid profile, blood pressure, and biomarkers of inflammation and oxidative stress. These outcomes were selected because they encompass both direct prognostic indicators (mortality) and key modifiable risk factors and underlying mechanisms (dyslipidemia, hypertension, chronic inflammation, and oxidative damage) that drive cardiovascular and renal disease (16) progression in this population. Further, we have examined the potential adverse effects of nut interventions on the concentrations of phosphorus and potassium.

## 2 Methods

This review was conducted in accordance with the Preferred Reporting Items for Systematic Reviews and Meta-Analyses (PRISMA) reporting guidelines (17). The review protocol was prospectively registered on the Prospective International Register of Systematic Reviews (PROSPERO, Registration n^o^ CRD42024543880).

### 2.1 Search strategy and eligibility criteria

We conducted a comprehensive literature search of the Medline, EMBASE, Scopus, CINAHL, and Cochrane Library databases on 27 May 2024 and repeated on 13 February 2025 to identify studies reporting on nut consumption in patients with CKD. All databases were searched from inception date using the terms: Adult* AND (Diet OR Nuts OR “*Prunus Dulcis*” OR Almond* OR Anacardium OR Cashew* OR Corylus OR Hazelnut* OR Pistacia OR Pistachio* OR Juglans OR Walnut* OR Carya OR Pecan* OR Arachis OR Peanut* OR Pinus OR ‘Pine nut*’ OR Bertholletia OR ‘Brazil nut*’) AND (Chronic Renal Insufficiency* OR Chronic Renal Insufficiencies* OR Chronic Kidney Insufficiency* OR Chronic Kidney Insufficiencies OR Chronic Kidney Diseases* OR Chronic Kidney Disease* OR Chronic Renal Diseases* OR Chronic Renal Disease*). The complete search strategy for each database is described in Supplementary Table 1.

Inclusion and exclusion criteria were formed based on the PICOS (population, intervention, comparison, outcome, and study design) format, described in Table 1. We included articles that assessed nut intake in adults with CKD and were published in any language. Additionally, we evaluated changes in phosphorus and potassium levels as adverse effects of nut consumption in CKD. Excluded articles referred to studies that assessed individuals without CKD, did not have a comparative group, did not quantify dietary nut intake or combined nuts with other foods (e.g., seeds), or did not report on CKD-related outcomes. To complement the electronic search, reference lists from selected studies and reviews were manually examined.

**Table 1:** PICOS format structured research question.

PICOS elements	Inclusion criteria
Population	Adults (18 years or older) of both sexes diagnosed with CKD
Intervention	Nut consumption (whole nut, oil or extract of at least one of the following: almond, baru almond, Brazil nut, cashew, hazelnut, macadamia, peanut, pecan, pine nut, pistachio, walnut)
Comparison	Standard care without nuts or the lowest percentile of nut exposure
Outcome	Lipid profile, inflammatory markers, oxidative stress markers, blood pressure, and mortality
Study design	Cross-sectional, case–control, controlled trials (randomized or non-randomized) and cohort studies

### 2.2 Study screening and data extraction

The study screening process was conducted in two stages by two independent authors (TL and RSB) using the systematic review software Rayyan (Cambridge, United States) (18), which automatically identified and excluded duplicate articles. First, titles and abstracts were screened; secondly, the full text of the remaining articles was screened according to the pre-specified inclusion. A third author (MFM) settled disagreements on study screening.

Upon completion of screening, data were independently extracted from each article by two authors (TL and RSB) using a data extraction template on Microsoft Excel. Data collected included first author, year of publication, country in which the study was conducted, study design, participant characteristics (age and gender), type and quantity of nuts consumed, methodology used to assess nut intake, length of the intervention/follow-up, and the health outcomes measured.

### 2.3 Risk of bias assessment

The risk of bias assessment of eligible articles was performed independently by two authors (RSB and TL). Disagreements were resolved in consultation with a third author (MFM). The Cochrane Risk of Bias 2.0 (RoB 2) tool was used to assess the quality of randomized and crossover trials (19). Studies were determined as having “low risk,” “high risk” or “some concerns.” The Risk of Bias In Non-randomized Studies - of Interventions (ROBINS-I) tool was used to assess the risk of bias in observational studies and non-randomized trials. As per the ROBINS-I tool, the studies were identified as providing “low,” “moderate,” “serious,” “critical,” or “no information” for each of the seven domains (confounding; participants selection; classification of interventions; deviations from intended interventions; missing data; measurement of outcomes; selection of the reported result). A final judgment based on the domains was then made to determine the study as having “low,” “moderate,” “serious,” or “critical” risk of bias (20) (Supplementary Figures 1–3).

## 3 Results

### 3.1 Study selection

**Figure 1:**
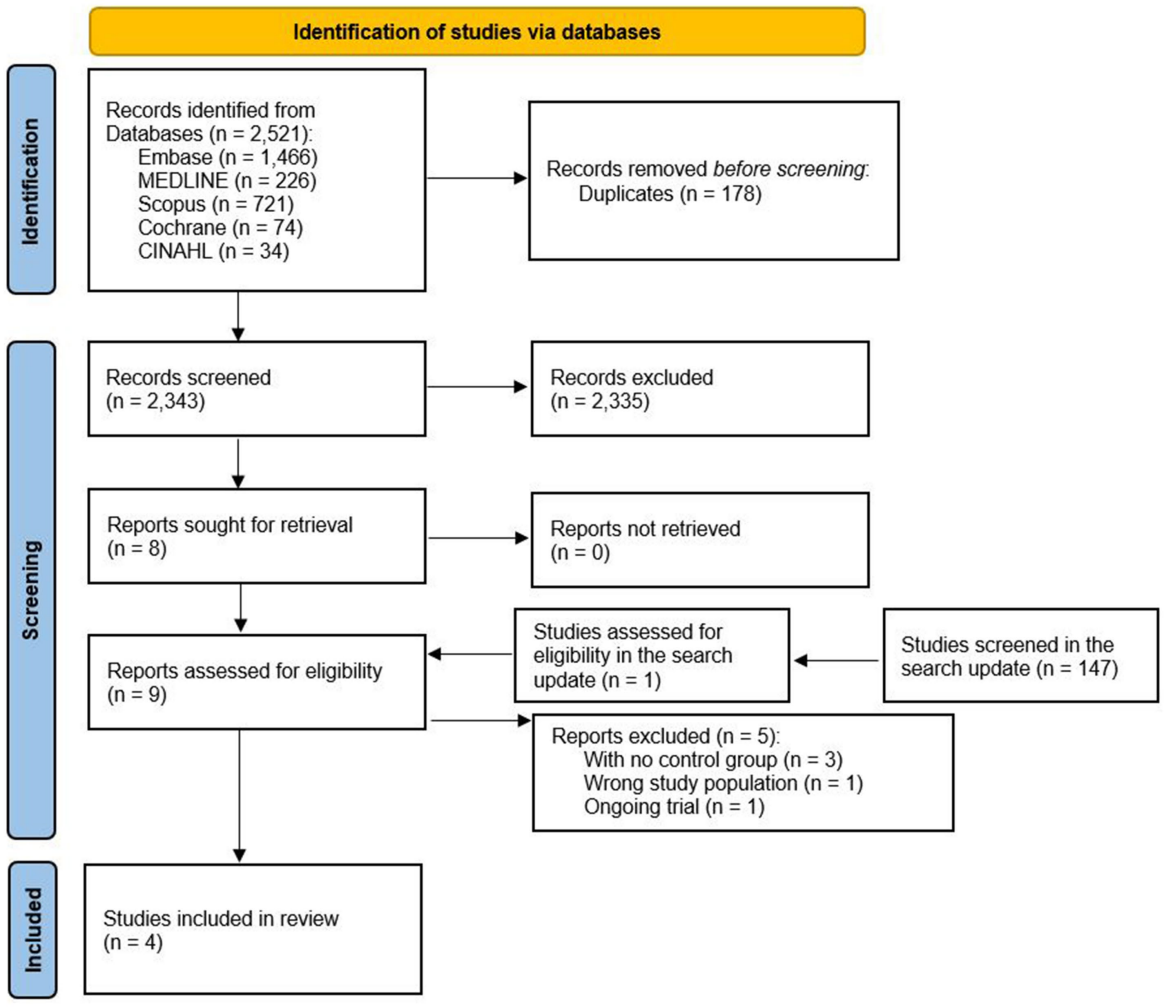
Flowchart of the article selection process.

The initial search identified 2,521 records, and after the exclusion of duplicates (*n* = 178), a total 2,343 articles were assessed for eligibility. In the first screening step, where abstracts and titles were screened, 2,335 records were excluded, and 8 full texts were comprehensively screened for eligibility. Additionally, one full-text article identified in the search update was also assessed for eligibility. Of these, five trials were excluded because of the absence of a control group (*n* = 1) wrong study population (*n* = 1), and unfinished study (*n* = 1). Thus, this systematic review included a total of four articles involving 1,270 participants (one cohort study (21) and three trials (22–24)) (Figure 1).

### 3.2 Study characteristics

This review included two RCTs (one had a parallel arm (22), and one had a crossover design (23)), one pilot trial (24), and one cohort study (21). Out of the 1,270 participants included in the studies reviewed, 1,203 were part of the cohort study. Two trials were conducted with patients receiving hemodialysis treatment (22, 24), and Sanchis et al. (23) focused on patients receiving conservative management (without dialysis), while Wang et al. (21) did not report whether participants were receiving dialysis. While the observational study reported exposure to different nut types without specifying them (21), the trials supplemented baru almond oil (22), walnuts (23), or Brazil nuts (24). The duration of intervention in the trials ranged from 30 days (walnut study) (23) to 3 months (Brazil nut study; baru almond oil: 12 weeks) (24). Two studies assessed oxidative stress and inflammatory markers (22, 24), and lipid profile (22, 23), while arterial pressure (23), and mortality (21) were assessed. Given the heterogeneity of study designs, types of nuts consumed, and outcome measures reported in the studies included in this review, a meta-analysis of study results was not feasible; therefore, this review focuses on a narrative synthesis of study outcomes. The number of participants in each study, the interventions/exposures, and the outcomes are shown in Table 2 (21–24).

**Table 2:** Characteristics of studies that assessed the effects of nut consumption in patients with CKD.

First author (year), country or origin	Study design, length of intervention/follow-up	Participants, number, mean age (y), gender	Type and amount of nut intake	Comparison group	Outcome	Findings
Cardozo et al. (2016), Brazil (24)	Parallel pilot clinical trial, 3 months	25 participants receiving hemodialysis, treatment group mean age: 57.1 ± 12.0 y, 53.8% female; control group mean age: 52.0 ± 15.5 y, 75.0% female	1 Brazil nut/d (n = 13)	Brazil nut-free diet (*n* = 12)*	NRF-2 (gene expression)	Change (mean): Brazil nut: 0.76; *p* = 0.001; control: −0.08; *p* = 0.77
					NQO1 (gene expression)	Change (mean): Brazil nut: 1.43; *p* = 0.001; control: −0.28; *p* = 0.63
					NF-kB (gene expression)	Change (mean): Brazil nut: −1.06; *p* = 0.001; control: −0.20; *p* = 0.09
					IL-6 (pg/dL)	Change: Brazil nut (mean): −0.37; *p* = 0.03; control (median): −2.28; *p* = 0.26
					MDA (nmol/mL)	Change (mean): Brazil nut: −6.7; *p* = 0.01; control: −2.8; *p* = 0.47
					CRP (ng/dL)	Change (mean): Brazil nut: −1.9; *p* < 0.05; control: −1.5; *p* = 0.60
Schincaglia et al. (2020), Brazil (22)	Parallel RCT, 12 weeks	29 participants receiving hemodialysis, treatment group mean age: 49.3 ± 3.4 y, 33.3% female vs. comparison group mean age: 51.3 ± 3.0 y, 35.3% female	5 g Baru almond oil/d (*n* = 12)	Placebo (*n* = 17)	CRP (mg/L)	Change (mean): baru oil: −1.1; placebo: 0.8; Treatment effect: *p* = 0.04
					SOD (U/g protein)	No difference
					Catalase (mmol/g protein)	No difference
					MDA (mmol/g protein)	No difference
					TC, TG, HDL, LDL, VLDL, nHDL, (mg/dL)	No difference
					Plasma fatty acid composition	No difference
Sanchis et al. (2019), Spain (23)	Crossover RCT, 30 days	13 participants with moderate to severe CKD (stage 3 or 4) with no renal replacement therapy, median age: 71 y interquartile range: 66–77 y, 46.2% female	Walnut: 30 g walnuts/d (*n* = 13)	Control: 5 g olive oil/d (*n* = 13)	Systolic blood pressure (mm/Hg)	Change [median (Q1, Q3)]: walnut: −4.0 (−28.0,0.0); control: 5.0 (−10.0,13.0); Intergroup difference: *p* = 0.040
					LDL, HDL, TC, TG (mg/dL)	No difference
Wang et al. (2022), China (21)	Cohort, 11 y (mean follow-up time)	6,072 adults (1,203 with CKD), Groups divided by frequency of nut consumption	CKD patients	CKD patients: non-nut consumers (*n* = 185)	All-cause mortality	1–11 times/y: *β*: 0.91; 95%CI: 0.70–1.18 1–3 times/month: *β*: 0.94; 95%CI: 0.72–1.22 1–6 times/wk.: *β*: 0.63; 95% CI: 0.47–0.86 >Once/day: *β*: 0.79; 95%CI: 0.46–1.37.
		Non-consumers: 51.0 (30.0–71.0) y, 52.8% female	1–11 times/ year (*n* = 368)		CVD mortality	No difference
		1–11 times/year: 47.0 (32.0–65.0) y, 53.9% female	1–3 times/month (*n* = 334)			
		1–3 times/month: 48.0 (34.0–65.0) y, 53.7% female	1–6 times/week (*n* = 270)			
		1–6 times/week: 54.0 (40.0–68.0) y, 52.0% female	> once a day (*n* = 47)			
		> than once a day: 61.0 (46.3–71.0) y, 46.7% female				

CKD, chronic kidney disease; CRP, C-reactive protein; NQO1, NAD(P)H quinone oxidoreductase 1; NF-kB, nuclear factor kappa B; IL, interleukin; MDA, malondialdehyde; SOD, superoxide dismutase; TC, Total Cholesterol; TG, Triglycerides; HDL, High-Density Lipoprotein; LDL, Low-Density Lipoprotein; VLDL, Very Low-Density Lipoprotein; nHDL, Non-HDL Cholesterol; CVD, cardiovascular disease; RCT, randomized controlled trial; y, year; wk, week. *No between-groups comparison, only within-groups comparison reported.

### 3.3 Risk of bias

The study by Cardozo et al. (24) was judged to have a “high” risk of bias, as it did not provide a direct comparison between intervention and control groups, but only reported within-group pre–post analyses. Wang et al. (21) was determined to have “moderate” risk due to confounding bias caused by the intrinsic characteristics of the cohort participants (Supplementary Figure 1). According to the Cochrane RoB 2 tool, the study by Sanchis et al. (23) was rated as having “some concerns” because it was an open-label study and intention-to-treat analysis was not performed (Supplementary Figure 2). The study by Schincaglia et al. (22) was determined as having “high risk” due to the lack of information about randomization or allocation method, missing intention-to-treat analysis and significant loss during the follow-up (more than 30%) (Supplementary Figure 3).

### 3.4 Oxidative stress and inflammatory markers

Two studies assessed the effects of nuts on inflammatory and oxidative stress markers in hemodialysis patients (22, 24). In a pilot trial, Cardozo et al. (24) reported that the consumption of one Brazil nut a day for 3 months increased the expression of nuclear factor E2-related factor 2 (Nrf-2) and NAD(P)H: quinone oxidoreductase 1 (NQO1), and decreased the expression of nuclear factor kappa B (NF-kB) in relation to baseline. Among oxidative stress and inflammation markers, Brazil nut supplementation reduced plasma malondialdehyde (MDA), CRP and interleukin-6 (IL-6), while no significant changes were observed in the control group. However, it is important to note that statistical analyses were primarily conducted within each group (pre–post comparison) rather than directly testing between-group differences, which increases the risk of confounding and limits causal inference. In the study by Schincaglia et al. (23), supplementation with baru almond oil (5 g/day) for 12 weeks decreased CRP but had no effect on MDA or the antioxidant enzymes superoxide dismutase (SOD) and catalase (CAT) compared to a placebo (22).

### 3.5 Lipid profile and blood pressure

The effects of nut intake on plasma lipid concentrations were assessed in two studies. Interventions with baru almond oil (22) or walnuts (23) had no effects on blood lipids. In the only study assessing the effects of nuts on blood pressure, Sanchis et al. (23) demonstrated that 30 g walnuts/d for 30 days significantly reduced systolic blood pressure in comparison to the control group.

### 3.6 Mortality

Only one study examined the association between nut intake and mortality. In their cohort study with a mean follow-up of 11 years, Wang et al. (21) analyzed data from 1,203 US adults with CKD to investigate the association between frequency of nut consumption (total nuts with no distinction of nut type) and cardiovascular and all-cause mortality. Overall, nut consumption was not associated with cardiovascular mortality, but consuming nuts 1–6 times/week was significantly associated with lower all-cause mortality rates (HR: 0.63, 95%CI: 0.47–0.86; *p* = 0.03; model adjusted for age, gender, ethnicity, glomerular filtration rate and urinary albumin-to-creatinine ratio, dietary intake of energy, carbohydrates, sugar, fats, phosphorus, sodium and potassium, alcohol consumption, smoking status, obesity, blood lipids, serum potassium and phosphorus, and health status [hypertension, diabetes, coronary heart disease, congestive heart failure, stroke, cancer], with no observed benefits seen for the consumption of more than once a day.

### 3.7 Adverse effects

Two studies evaluated serum phosphorus and potassium levels after walnut consumption in CKD patients (22, 23) and found no significant changes in these parameters, indicating that walnut intake is safe regarding these electrolytes balance.

## 4 Discussion

This systematic review examined the effects of nut consumption on oxidative stress, inflammatory biomarkers, and mortality in patients with CKD. Despite broad inclusion criteria that encompassed different study designs, only four studies met eligibility for inclusion. Collectively, these studies suggest a potential role for nuts in CKD management, with reported benefits on oxidative stress, inflammatory markers, and all-cause mortality. However, most studies were judged to have a high risk of bias, and considerable heterogeneity was observed, limiting the strength of the evidence and highlighting an important gap in the literature regarding the potential benefits of nuts for this population.

Nuts have an optimal fatty acid profile, with a high concentration of polyunsaturated and monounsaturated fatty acids. Additionally, nuts are a rich source of proteins, fibers, B-vitamins, minerals and polyphenols (5, 8, 25). Nuts have been incorporated into several healthy diets, such as the Mediterranean diet (26), the global planetary health diet (27) and several other plant-based diets (28). There is a growing body of evidence associating the consumption of these diets with lower rates of cancer, cardiovascular disease, obesity, and mortality (29–33). In CKD patients, however, despite a lack of robust RCTs, epidemiological studies suggest the same benefits of whole-food plant-based diets in cardiovascular health and CKD progression (34–36). Whole-food plant-based diets constitute a shift away from the previously accepted diet for CKD that is low in protein (0.55–0.60 g/kg/day for nondiabetics and 0.60–0.80 g/kg/day for those with diabetes), high in fruits and vegetables (with consideration of potassium and phosphorous content to avoid elevated levels of these electrolytes), and low in sodium (<2.3 g/day) (4). Thus, to restrict the intake of protein, phosphorus, and potassium in order to meet the dietary guidelines, CKD patients commonly eat a limited amount of healthy ingredients such as fruits, vegetables, and nuts. However, encouraged by the benefits of healthy diets observed in recent epidemiological studies, some recent studies have assessed the effects of nut consumption in CKD patients, which justifies the fact that the trials included in this review were all recently published. Our review identified two studies showing that walnut intake did not alter serum phosphorus or potassium concentrations (22, 23), suggesting that walnuts do not pose a risk of electrolyte imbalance in patients with advanced CKD receiving conservative treatment. However, further research is needed to determine whether similar effects occur with other types of nuts.

Inflammation and oxidative stress are non-traditional risk factors for CKD development and progression (3). They result from dysregulation caused by an imbalance between reactive oxygen and nitrogen species, and antioxidant systems, prompting the release of pro-inflammatory cytokines (3, 37). These processes are regulated by two transcriptional factors, NF-κβ and Nrf2, the former induced when oxidative stress is high and resulting in a pro-inflammatory state, and the latter is responsible for mitigating inflammation and oxidative stress (37). These risk factors are even more critical in patients with kidney injury. The kidneys are metabolically active organs, making them more vulnerable to damage caused by oxidative stress (38). Further, several uremic toxins are associated with increased oxidative stress in CKD (38). In addition, the increased production of proinflammatory cytokines and oxidative stress mediated by the activation of macrophages and monocytes constitutes the fundamental basis for initiating the inflammatory cascade, worsening renal function (39). The two trials evaluating nuts and their effects on oxidative stress and inflammation found that interventions with both Brazil nuts and baru almond oil reduced CRP, a marker of acute inflammatory response (22, 24). In addition, Brazil nut consumption was associated with reduced NF-κB expression and increased expression of NRF-2 and NQO1, alongside lower IL-6 concentrations, indicating a more favorable inflammatory profile (24). Brazil nuts also reduced MDA levels, a marker of oxidative stress, whereas baru almond oil showed no effect on MDA or on the antioxidant enzymes SOD and catalase. Given the differences in interventions and outcomes assessed, the heterogeneity between these two studies limits firm conclusions. Nevertheless, the findings suggest a potential benefit of nut consumption for oxidative stress and inflammation in CKD, warranting further investigation. In addition to the bioactive compounds found in all the different nut types, Brazil nuts are the richest food source of selenium, an essential micronutrient for humans and animals with a key role in thyroid function, immune response, and maintenance of cell redox balance through its presence in selenoproteins (40). Previous research has demonstrated a clear effect of Brazil nuts in enhancing the antioxidant status in different study populations, such as people with obesity, dyslipidemia, type 2 diabetes, as well as so-called healthy individuals (41).

The consumption of nuts has been associated with reduction of all-cause mortality in several studies, summarized in an umbrella review which identified that a daily consumption of 28 g of nuts was associated with 22% reduction in all-cause mortality in CKD-free cohorts (42). It appears that the protective effects of nuts on all-cause mortality is also observed in CKD patients as reported by Wang et al. (21). Nonetheless, we highlight that besides being the only study this far to investigate such association, it has several limitations such as a lack of information on the types and amounts of nuts consumed. Considering that CKD contributes to over 1.43 million deaths per year (1, 2), we highly encourage future studies to investigate the long-term effects of nuts on mortality in this population.

The limitations of this systematic review, which includes only four studies, highlights the need for additional research to better understand the potential benefits of adding nuts to the diet for improving the prognosis of patients with CKD. The studies included in this review used different nut types and had small sample sizes, which hinders generalization of findings across all nut types. Further, we acknowledge that all the four studies papers in this systematic review presented moderate-to-high risk of bias, compromising the quality of evidence provide. Even though we found that overall nut consumption seems to be safe for patients with CKD in terms of electrolytes balance, we acknowledge that further studies investigating the different nut types with larger sample sizes are necessary for the translation of evidence into clinical practice on the management of CKD patients.

## 5 Conclusion

Although the small trials included in this systematic review suggest that nuts might have the potential to mitigate non-traditional risk factors for CKD progression and thus improve the prognosis of people with this chronic condition, lack of high-quality trials involving nut intake as a strategy for CKD management hinders clear evidence. Thus, we highlight the necessity for more trials using nuts in CKD treatment in order to translate the findings into clinical practice.

## Data Availability

The original contributions presented in the study are included in the article/Supplementary material, further inquiries can be directed to the corresponding author/s.
