# Supplementary material for: Nut consumption in chronic kidney disease: a systematic review
Source: Front Nutr. 2025 Nov 10;12:1659516. doi: 10.3389/fnut.2025.1659516 (PMC12642863; doi:10.3389/fnut.2025.1659516)

**Supplementary Table 1. Search strategy**

| **Database** | **Search strategy** |
| --- | --- |
| MEDLINE (PubMed) | ((Chronic Renal Insufficiency OR Chronic Renal Insufficiencies OR Chronic Kidney Insufficiency OR Chronic Kidney Insufficiencies OR Chronic Kidney Diseases OR Chronic Kidney Disease OR Chronic Renal Diseases OR Chronic Renal Disease) AND (Nut OR nuts OR Juglans OR Walnut OR Walnuts OR Juglans regia OR Juglans nigra OR Anacardium OR Anacardiums OR Cashew OR Cashews OR Prunus dulcis OR Prunis communis OR Prunus amygdalus OR Almonds OR Almond OR Corylus OR Filbert OR Filberts OR Hazelnuts OR Hazelnut OR Pistacia OR Pistachio OR Pistachios OR Pistacia lentiscus OR Carya OR Caryas OR Pecans OR Pecan OR Carya illinoensis OR Carya illinoenses OR Arachis OR Arachis hypogaea OR Peanuts OR Peanut OR Pinus OR Pinus pinaster OR Pinus abies OR Pinus tremula OR Pinus radiata OR bertholletia or Bertholletias OR Brazil Nuts OR Brazil Nut OR Bertholletia excelsa OR Bertholletia excelsas)) |
| EMBASE | 'chronic kidney failure'/exp OR 'chronic kidney failure' AND 'nut'/exp OR nut OR 'nut consumption'/exp OR 'nut consumption' OR 'walnut'/exp OR walnut OR juglans OR 'anacardium'/exp OR anacardium OR 'cashew nut'/exp OR 'cashew nut' OR 'almond'/exp OR almond OR 'prunus dulcis' OR 'hazelnut'/exp OR hazelnut OR Corylus OR 'pistacia'/exp OR Pistacia OR 'pistachio'/exp OR pistachio OR 'carya'/exp OR carya OR 'pecan'/exp OR pecan OR 'arachis'/exp OR arachis OR 'peanut'/exp OR peanut OR 'pine'/exp OR pine OR 'bertholletia'/exp OR bertholletia OR 'brazil nut'/exp OR 'brazil nut' |
| SCOPUS | (ALL(nut OR 'nut AND consumption' OR walnut OR juglans OR anacardium OR 'cashew AND nut' OR almond OR 'prunus AND dulcis' OR hazelnut OR corylus OR pistacia OR pistachio OR carya OR pecan OR arachis OR peanut OR pine OR bertholletia OR 'brazil AND nut') AND ALL('chronic AND kidney AND failure')) |
| CINAHL | chronic kidney failure OR Chronic Renal Insufficiency OR Chronic Renal Insufficiencies OR Chronic Kidney Insufficiency OR Chronic Kidney Insufficiencies OR Chronic Kidney Diseases OR Chronic Kidney Disease OR Chronic Renal Diseases OR Chronic Renal Disease AND nut OR nut consumption OR walnut OR juglans OR anacardium OR cashew nut OR almond OR prunus dulcis OR hazelnut OR corylus OR pistacia OR pistachio OR carya OR pecan OR arachis OR peanut OR pine OR bertholletia OR brazil nut |
| CENTRAL - COCHRANE | ‘’ chronic kidney failure’’ OR ‘’Chronic Renal Insufficiency’’ OR ‘’Chronic Renal Insufficiencies’’ OR ‘’Chronic Kidney Insufficiency’’ OR ‘’Chronic Kidney Insufficiencies’’ OR ‘’Chronic Kidney Diseases’’ OR ‘’Chronic Kidney Disease’’ OR ‘’Chronic Renal Diseases’’ OR ‘’Chronic Renal Disease’’  nut OR '’nut consumption'’ OR walnut OR juglans OR anacardium OR '’cashew nut’' OR almond OR ‘'prunus dulcis’' OR hazelnut OR corylus OR pistacia OR pistachio OR carya OR pecan OR arachis OR peanut OR pine OR bertholletia OR '’brazil nut'’ |

**Supplementary Figure 1 - Risk of bias results for included quasi-experimental studies according to the Risk of Bias in Nonrandomized Studies–of Interventions (ROBINS-I) tool.**


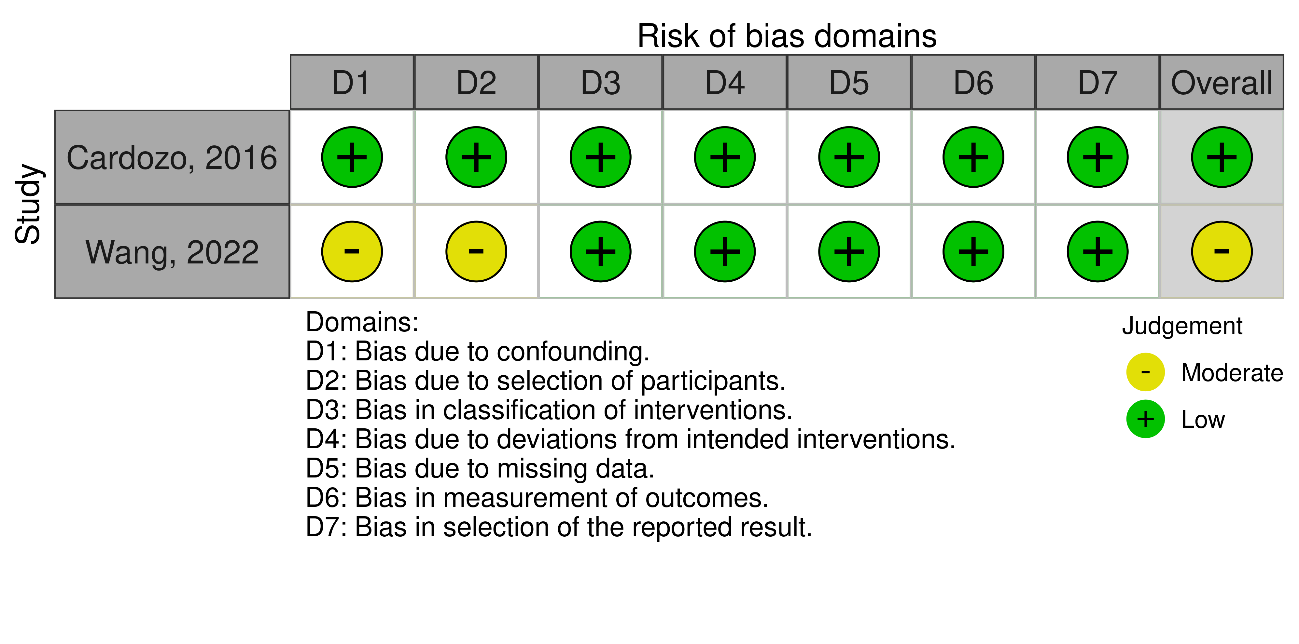


**Supplementary Figure 2 - Risk of bias results for included cross-over studies according to the Cochrane Risk of Bias 2 (RoB 2) tool.
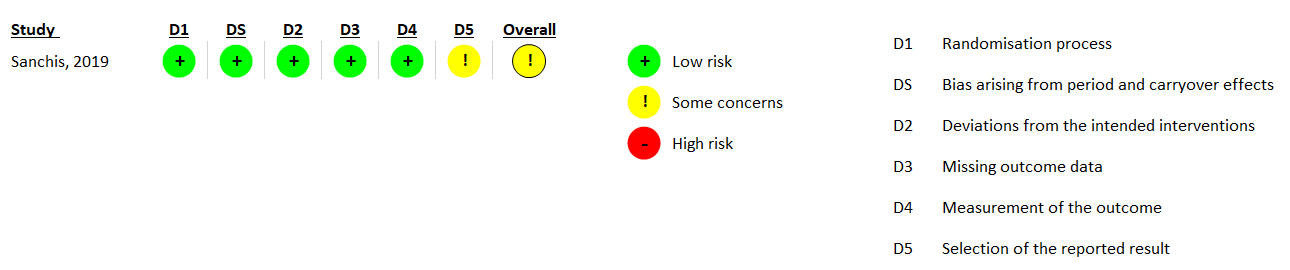
**

**Supplementary Figure 3 - Risk of bias results for included randomized controlled trials according to the Cochrane Risk of Bias 2 (RoB 2) tool.**


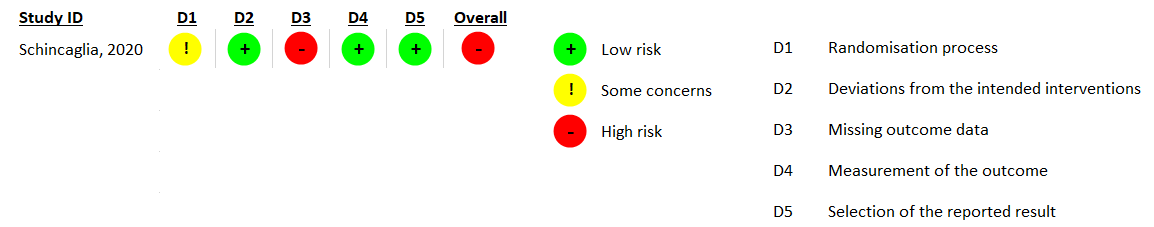

Supplement: Supplementary file 1 [file Table_1.docx]
